# Focal segmental glomerulosclerosis and end-stage kidney disease in children

**Published:** 2015-05-17

**Authors:** Alaleh Gheissari

**Affiliations:** Department of Pediatric Nephrology, Isfahan University of Medical Sciences, Isfahan, Iran

**Keywords:** Focal segmental glomerulosclerosis, End stage renal disease, Children

**Implication for health policy/practice/research/medical education:**

Focal segmental glomerulosclerosis (FSGS) is one of the principal causes of end-stage kidney disease in children worldwide. During the past decades, different genes mutations have been implicated in pathogenesis of resistant sporadic and hereditary familial forms of FSGS. Nephrin, podocin, alpha-actinin 4 (ACTN4) are some of podocyte structural proteins that their role in pathogenesis of FSGS have already been discussed. However, the role of TRPC6, a transient receptor potential ion channel, in hereditary forms of FSGS has been discovered. Regarding the roles of TRPC6 channels in proteinuria new trends to use TRPC6 blocking agents though challenging are developed.

Focal segmental glomerulosclerosis (FSGS) is one of the principal causes of end-stage kidney disease in children worldwide (1-3). During the past decades, different genes mutations have been implicated in pathogenesis of resistant sporadic and hereditary familial forms of FSGS. Nephrin, podocin, alpha-actinin 4 (ACTN4) are some of podocyte structural proteins that their role in pathogenesis of FSGS have already been discussed. However, the role of TRPC6, a transient receptor potential ion channel, in hereditary forms of FSGS has been discovered for the first time by Winn et al (4). TRP channels have been involved in H_2_O_2_-induced Ca^2+^ influx and cell death and degenerative disease (5).

Many published papers focused on the role of TRPC6 mutation in hereditary forms and adult onset types of the disease.

A large recent cohort study by Santín et al showed TRPC6 gene mutation in both late onset familial and non-familial FSGS (6). They found TRPC6 gene mutation in 2 of 130 Spanish patients. Nonetheless, one case of familial type FSGS with TRPC6 gene mutation was reported.

Studies on familial forms of FSGS showed novel gene mutations. Mottl et al reported a novel frameshift mutation in TRPC6, D873fsX878 in a 35 years old female that had a history of asymptomatic nephrotic range proteinuria during pregnancy and same mutation in her mother (7). Accordingly, Hofstra et al investigated mutations of all 13 exons of TRPC6 in 5 patients of 5 different Dutch families with autosomal dominant FSGS. None of the known mutations of TRPC6 was found. Nevertheless, a novel c.524G>A sequence variant resulting in a p.Arg175Gln (R175Q) substitution in the TRPC6 protein was identified in one family (8). The positive results of studies on patients with early onset FSGS and significant role of TRPC6 channels in podocyte disease influenced many researchers to investigate TRPC6 gene mutation in childhood resistant FSGS. Likewise, Gigante et al analyzed TRPC6 gene mutations in 33 Italian children with sporadic early onset FSGS and three families with adult-onset type of FSGS (9). Three heterozygous missense mutations (c.374A_G_p.N125S, c.653A_T_p.H218L, c.2684G_T_p.R895L) were identified in a 18 years old boy with child-onset FSGS, a 2 years old girl with collapsing type of FSGS, and two siblings with steroid-resistant nephritic syndrome (4 and 14 years old), respectively. Furthermore, Lipska et al screened autosomal dominant cases among 227 patients aged 10-20 years with non-syndromic steroid resistant nephrotic syndrome for WT1, TRPC6, ACTN4, and INF2 mutations (10). However, in none of them TRPC6 gene mutation was reported.

Missense mutation was detected in sporadic and familial cases of steroid resistant nephritic syndrome in Turkish children (11). They found a variant (L395A) in one patient, intronic nucleotide substitution (c.171 1 16 A>G and c.171 1 86 G>C) in 6 patients and missense (A404V; rs36111323) and synonymous (N561N; rs12366144) amino acid variants in 9 patients.

In this regard, Mahesh-Kumar et al analyzed TRPC6 gene mutation among Indian children with steroid resistant nephrotic syndrome (12). A total 49 unrelated children aged less than 10 years were recruited. The authors used PCR-RFLP to genotype two candidate SNPs in the promoter region of TRPC6 gene. Nevertheless, no mutation was reported among patients.

However, the controversy among results of studies on gene mutation analysis on steroid resistant nephrotic syndrome does not undermine the significant role of TRPC6 gene mutations in producing podocytopathy and steroid resistant nephritic syndrome. Different biological functions such as mechanosensation, cell growth and vasoregulation are intervened by TRP channels (13,14).

Mutation in TRPC6 not only leads to abnormalities in the slit diaphragm but also amplifies injurious signals mediated by angiotensin II (2). The exact mechanism of FSGS through enhancing intracellular calcium influx into the podocyte is not precisely described (2). Altering contractile structure of foot process and changing ultrafiltration coefficient have been described as the responsible mechanisms (2). In addition, disruption of the slit diaphragm architecture may cause overexpression and mislocalization of TRPC6 in podocytes (15).

Regarding the roles of TRPC6 channels in proteinuria new trends to use TRPC6 blocking agents though challenging are developed (2).

## Authors’ contribution

AG was the single author of the paper.

## Conflicts of interest

The author declared no competing interests.

## Ethical considerations

Ethical issues (including plagiarism, data fabrication, double publication) have been completely observed by the author.

## Funding/Support

None.
